# Antibiofilm potential of plant extracts: inhibiting oral microorganisms and *Streptococcus mutans*

**DOI:** 10.3389/fdmed.2025.1535753

**Published:** 2025-04-04

**Authors:** Nomi Bartels, Aikaterini Argyropoulou, Ali Al-Ahmad, Elmar Hellwig, Alexios Leandros Skaltsounis, Annette Wittmer, Kirstin Vach, Lamprini Karygianni

**Affiliations:** ^1^ Department of Prosthodontics, Faculty of Medicine Carl Gustav Carus, TU Dresden, Dresden, Germany; ^2^ Department of Pharmacognosy and Natural Products Chemistry, Faculty of Pharmacy, National and Kapodistrian University of Athens, Athens, Greece; ^3^ Department of Operative Dentistry and Periodontology, Medical Center, Faculty of Medicine, University of Freiburg, Freiburg, Germany; ^4^ Institute of Medical Microbiology and Hygiene, Faculty of Medicine, University of Freiburg, Freiburg, Germany; ^5^ Institute for Medical Biometry and Statistics, Faculty of Medicine and Medical Center, University of Freiburg, Freiburg, Germany; ^6^ Clinic of Conservative and Preventive Dentistry, Center of Dental Medicine University of Zurich, Zurich, Switzerland

**Keywords:** Mediterranean herb extracts, oral mouthwashes, antimicrobial activity, biofilm inhibition, *Streptococcus mutans*

## Abstract

**Introduction:**

A range of disinfectant mouthwashes are available for oral hygiene. The gold standard is Chlorhexidine digluconate (CHX), which, like other available products, cannot be used without side effects in the long term. However, in recent years, therapy with herbal products, often considered antiquated, has regained considerable interest. Therefore, the search for plant compounds as an alternative to existing oral disinfectants is meaningful.

**Methods:**

In this study, eleven Mediterranean plant extracts were tested for their antimicrobial effect *in vitro*. Methanol extracts of the following plants were produced by the pharmaceutical faculty of the University of Athens: *Mentha aquatica*, *Mentha longifolia*, *Sideritis euboea*, *Sideritis syriaca*, *Stachys spinosa*, *Satureja parnassica*, *Satureja thymbra*, *Lavandula stoechas*, *Achillea taygetea*, *Phlomis cretica*, and *Vaccinium myrtillus*. The extracts were dissolved for microdilution experiments at concentrations ranging from 10 to 0.019 mg/ml. The oral pathogens tested were *Streptococcus mutans*, *Streptococcus oralis*, *Streptococcus sobrinus*, *Prevotella intermedia*, *Fusobacterium nucleatum*, *Parvimonas micra*, *Porphyromonas gingivalis*, and *Candida albicans*. *Enterococcus faecalis*, *Staphylococcus aureus*, and *Escherichia coli* were used as references.

**Results:**

All extracts, except the methanol extract of *V. myrtillus*, showed an antibacterial effect at concentrations ranging from 10 to 0.15 mg/ml. None of the extracts exhibited a significant antifungal effect. In general, the anaerobic pathogens could be inhibited and killed at lower concentrations compared to the aerobic pathogens. *S. oralis* also showed good susceptibility to the extracts. Additionally, the extracts' ability to inhibit biofilm formation by *S. mutans* was tested. *L. stoechas* at a concentration of 0.3 mg/ml showed a moderate inhibitory effect. The extracts of *L. stoechas*, *S. thymbra*, *S. parnassica*, and the methanol extract of *V. myrtillus* were effective at concentrations up to 1.25 mg/ml. *P. cretica* was able to inhibit and kill *S. mutans* at a concentration of 0.6 mg/ml, but its effectiveness in biofilm inhibition significantly decreased at 2.5 mg/ml.

**Discussion:**

The study's hypothesis that all extracts would exhibit an antimicrobial effect was thus confirmed.

## Introduction

A quarter of all medical prescriptions consist of drugs based on substances derived from plants or synthetic analogues derived from plants (1). Particularly in developing countries, plant-based drugs form the foundation of healthcare. Traditional medicine encompasses over 20,000 plant species, all of which hold potential as sources for new medicines (2). The diversity of structural compounds in plants is immense, offering numerous chemical structures that could potentially be effective (3). Effective natural plant products may include secondary plant compounds, organic compounds, phytochemical components, or bioactive compounds. Prominent plant secondary compounds comprise alkaloids, terpenes, flavonoids, and phenols (4). Maintaining proper oral hygiene is crucial for preventing oral diseases and involves regular removal and prevention of biofilm formation. Alongside mechanical removal using toothbrushes and oral hygiene aids, mouthwashes are utilized to support daily oral hygiene practices (5).

The Mediterranean region is home to a rich diversity of flora, many of which have long been used in traditional medicine for their therapeutic properties. These plants are not only vital to the region's ecosystem but also play a crucial role in local economies, as they are sources of food, medicinal products, and essential oils.

Chlorhexidine digluconate (CHX) serves as the gold standard for disinfecting mouthwashes. A 0.2% CHX solution is commonly employed to prevent biofilm formation in the oral cavity. CHX, being cationic, interacts with the negatively charged bacterial surface, disrupting the cell membrane and leading to bacterial death (6). However, prolonged use of CHX can result in undesired side effects such as extrinsic discoloration of teeth, changes in mucosal membranes, temporary taste disturbances, and increased calculus formation (5). CHX, due to its non-selective toxicity towards bacterial cells, can also cause harm to bone or mucosal cells (7, 8). Therefore, there is a need to explore alternatives to CHX.

Natural antimicrobials, especially those derived from plant extracts, play a crucial role in reducing plaque accumulation by targeting the bacteria involved in biofilm formation. These natural extracts possess antimicrobial and anti-inflammatory properties, showing great promise in managing peri-implantitis by addressing plaque buildup and biofilm development, two key factors in the onset and progression of the disease. Compounds found in these extracts, such as flavonoids, polyphenols, and alkaloids, exhibit strong antimicrobial properties that can prevent bacterial adhesion and disrupt biofilm formation on teeth and gums (9, 10). For example, green tea polyphenols, particularly epigallocatechin gallate (EGCG), have been found to effectively inhibit bacterial growth and reduce plaque formation (11). Similarly, extracts from neem and licorice demonstrate antimicrobial effects that help limit plaque accumulation by interfering with bacterial cell adhesion and their metabolic processes (12, 13). By minimizing plaque buildup, these natural antimicrobials contribute to improved oral health and decrease the risk of developing periodontal disease. Incorporating these plant-based solutions into oral hygiene practices may provide a complementary approach to conventional dental care.

The effects of the extracts on Gram-positive and facultative anaerobic microorganisms, such as *Streptococcus mutans*, *Streptococcus sobrinus*, and *Streptococcus oralis*, were investigated. Additionally, the impact on Gram-negative and anaerobic pathogens including *Porphyromonas gingivalis*, *Prevotella intermedia*, and *Fusobacterium nucleatum*, as well as the Gram-positive anaerobic bacterium *Parvimonas micra* and the fungus *Candida albicans*, were assessed. Reference microorganisms consisted of the Gram-positive facultative anaerobic pathogens *Staphylococcus aureus* and *Enterococcus faecalis*, along with the Gram-negative facultative anaerobe *Escherichia coli*. In previous studies, medical devices in contact with *S. mutans* increased the risk of infection. A variety of treatment options have been applied to treat such infections (14–16). The use of herbal drugs may impact this interaction by reducing microbial load and promoting alternative therapies (17). The antibacterial properties of Mentha spp., *Sideritis* spp., *Lavandula* spp., *Satureja* spp., *Phlomis* spp., and *Stachys* spp. against diverse Gram-positive and Gram-negative microorganisms have been highlighted in various studies (18–22). However, their impact on a variety of oral microorganisms has yet to be elucidated.

The 11 Mediterranean plants were selected based on their traditional medicinal uses, availability, and previous evidence of antimicrobial properties. These plants are known to contain bioactive compounds, such as essential oils and phenolic compounds, which have demonstrated potential antimicrobial and biofilm-inhibitory effects. A wide concentration range (from 10 to 0.15 mg/ml) was tested to determine both antimicrobial efficacy (Minimum Inhibitory Concentrations, or MICs) and biofilm-inhibitory effects. This approach aligns with previous studies and ensures that both high and low activity thresholds are included for reliable *in vitro* testing.

The growing global dependence on plant-based compounds for medical applications highlights the importance of exploring their potential in managing oral health. With a quarter of all medical prescriptions based on plant-derived substances and over 20,000 plant species employed in traditional medicine, the structural diversity of bioactive compounds in plants presents significant opportunities for discovering new therapeutic agents (23–25). This is particularly relevant for tackling issues like plaque accumulation and biofilm formation, which are central to oral diseases such as peri-implantitis and periodontitis. Given the limitations of commonly used antimicrobial agents like chlorhexidine digluconate (CHX), which can cause side effects such as tooth discoloration, changes in mucosal tissues, and potential cytotoxicity, there is an increasing demand for natural alternatives that are both safer and equally effective (26, 27). Plant extracts, rich in secondary metabolites such as flavonoids, terpenes, and phenolic compounds, have shown antimicrobial and biofilm-inhibitory effects against a variety of microorganisms (28). These natural compounds can disrupt bacterial adhesion, inhibit biofilm formation, and reduce microbial load, making them promising candidates for oral hygiene applications (29).

This study aims to evaluate the antimicrobial and biofilm-inhibitory potential of selected Mediterranean plant extracts, focusing on their activity against a range of Gram-positive and Gram-negative oral microorganisms, including *Streptococcus mutans*, *Porphyromonas gingivalis*, and *Candida albicans*. By investigating the efficacy of these extracts at various concentrations, the study seeks to identify plant-based alternatives to conventional antimicrobials that could be integrated into oral care strategies, ultimately contributing to improved management of conditions related to oral biofilm. The objective of this study was to assess the antimicrobial potential of the provided plant extracts in order to identify extracts suitable for further *in vivo* investigations. The plants examined in this study are indigenous to the Mediterranean region. They include *Mentha longifolia* (mint), *Mentha aquatica* (mint), *Lavandula stoechas* (lavender), *Sideritis syriaca* (ironwort, mountain tea), *Sideritis Euboea* (ironwort, mountain tea), *Satureja parnassica* (savory), *Satureja thymbra* (savory), *Phlomis cretica* (Cretan Jerusalem Sage), and *Stachys spinosa* (hedgenettle), belonging to the *Lamiaceae* family. *Achillea taygetea* (yarrow) belongs to the *Asteraceae* family, and *Vaccinium myrtillus* (bilberries, blueberries) belongs to the *Ericaceae* family. This study was conducted with the hypothesis that all extracts, particularly at higher concentrations, would exhibit antimicrobial effects.

## Materials and methods

### Plant extracts

Plant materials from eleven distinct plant species were gathered from different locations within the Greek periphery. The selected plant species included: *Mentha longifolia* L., *Lavandula stoechas* L., *Sideritis syriaca* L., *Mentha aquatica* L., *Satureja thymbra* L., *Satureja parnassica* Heldr. & Sart. ex Boiss., *Phlomis cretica* C. Presl, Sideritis euboea Heldr., *Stachys spinosa* L., *Achillea taygetea* Boiss. & Heldr., and *Vaccinium myrtillus* L. In the case of *Vaccinium myrtillus*, the focus was on collecting the fruits, while for the remaining plants, the aerial components were collected.

### Extraction process

The collected plant specimens underwent thorough grinding (Allenwest-Eac ltd, Brighton and Hove, United Kingdom) to achieve finely homogeneous powders, which were then subjected to ultrasound-assisted extraction (UAE) (30, 31). This process utilized an Elma S 100H (Elmasonic, Elma Schmidbauer GmbH, Singen, Germany) instrument, with a solvent mixture of MeOH/Water 80:20, for an extraction duration of 15 min at room temperature. The ratio of plant material to solvent was maintained at 1/10 (w/v). To ensure comprehensive extraction, the procedure was repeated twice for each sample. Following extraction, the solvents were meticulously evaporated under reduced pressure utilizing a Buchi Rotavapor R-200 rotary evaporator, maintaining a temperature of 40°C, until dryness was achieved.

### High performance thin layer chromatography (HPTLC) analysis

To generate the fingerprinting profiles of the diverse extracts, a Camag HPTLC instrument setup was employed (32, 33). Solutions of the extracts were formulated by dissolving 10 mg of each extract in 1 ml of hydroalcoholic. For the application of plant extract samples onto Thin layer chromatography (TLC) plates measuring 20 cm × 10 cm (silica gel 60, F254, Merck), the Automatic TLC Sampler (ATS4, CAMAG, Muttenz, Switzerland) was utilized, controlled by the VisionCats 2.3 software platform (CAMAG, Muttenz, Switzerland). The TLC Sampler was configured according to standard parameters: 6 tracks with 8 mm bands, an 8 mm distance from the lower edge, 20 mm from both left and right edges, and a spacing of 10.4 mm between individual tracks. The applied volume for each sample was 8 μl. The ensuing development of plates was performed within an automatic development chamber (ADC2), adhering to established guidelines: 20 min of chamber saturation with filter paper, 10 min of plate conditioning at 33% relative humidity (using MgCl2), and a subsequent 5-min plate drying period. The mobile phase employed was dichloromethane/methanol/water (70:30:4; v/v/v) for polar extracts, while ethyl acetate, methanol/water/formic acid (50:10:7:1; v/v/v/v) containing highly polar substances was chosen for other extracts. Imaging at both 254 and 366 nm was captured using a Visualizer 2 Documentation System (CAMAG, Muttenz, Switzerland).

### Bacterial and fungal strains

Seven bacterial strains of the oral microflora and the yeast *Candida albicans* DSM 1386 (German Collection of Microorganisms and Cell Cultures) were tested. Reference microorganisms were *Enterococcus faecalis* ATCC 29212 and *Escherichia coli* ATCC 25923 found in the intestinal tract and *Staphylococcus aureus* ATCC 25923, a colonizer of the skin surface. The reference organisms were used to compare the oral inhibitory effect with the general antimicrobial activity. *Streptococcus sobrinus* DSM 20381, *Streptococcus mutans* DSM 20523, *Streptococcus oralis* ATCC 35037, *Enterococcus faecalis* ATCC 29212 and *Staphylococcus aureus* ATCC 25923 are facultative anaerobic Gram-positive bacteria. *Escherichia coli* ATCC 25922 is also facultative anaerobic but Gram-negative. The tested bacteria *Porphyromonas gingivalis* W 381, *Prevotella intermedia* ATCC 25611, *Fusobacterium nucleatum* ATCC 25586 and *Parvimonas micra* ATCC 23195 are obligate anaerobe pathogens. The bacterial strain *E. faecalis* T9 was isolated in the Department of Dental Conservation and Periodontology of the University Hospital Freiburg. After thawing of the pathogens, subcultures were created. The facultative anaerobic pathogens were cultivated on Columbia-blood agar plates (CBA) plates. *E. coli*, *S. aureus*, *E. faecalis* and *C. albicans* for 18 h at 37°C and humid heat, the streptococci for 24 h at 37°C humid heat and 5%–10% CO_2_. The subcultures of the obligate anaerobic bacteria were cultured on yeast-cystein blood Agar (HCB) plates under exclusion of oxygen for 48 h at 37°C anaerobic incubation chambers (Anaerocult® IS, Merck Chemicals GmbH, Darmstadt, Germany). Colonies of facultatively anaerobic bacteria and *C. albicans* were mixed in 0.9% NaCl up to a McFarland turbidity of 0.5 and 1 for *C. albicans*. The McFarland turbidity was tested by DensiCheck (BioMèrieux SA, Marcy-l'Étoile Frankreich). The obligate anaerobic bacteria were adjusted in Wilkens-Chalgren Anaerobe Broth (WCB, IMMH Freiburg) in a McFarland turbidity of 0,5. A comparable number of colony forming units (CFU) should be available per well of the microtiter plate. 5 × 10^5^ CFU of the facultative anaerobic bacteria, 5 × 10^6^ CFU of the obligate anaerobic bacteria and 5 × 10^4^ CFU of the fungus.

### Determination and evaluation of the minimum inhibitory concentration (MIC)

In shafts 2–12 of the microtiter plates, the corresponding nutrient medium was first presented. BBL™ Mueller Hinton II Broth (MHB; Becton Dickinson GmbH, Heidelberg, Deutschland) as culture medium of the facultatively anaerobic germs and *C. albicans*, sterile WCB for the obligate anaerobic germs. The plant extracts were dissolved in Dimethylsulfoxide (DMSO) and 1:10 in the respective Culture medium diluted. Thus, the initial concentration for all extracts was 10 mg/ml. The experiments were performed in duplicate. The 96 well micotiter plate was divided up as follows. In rows A-H; column 2–12, 100 µl culture medium were added according to the germ to be tested. In column 1 200 µl of the respective diluted extract were pipetted at the initial concentration of 10 mg/ml. Using a multichannel pipette, 100 µl of the first column were removed and mixed with the bouillon in the second column. 100 µl were taken from this column, halving the extract concentration. This procedure was used up to a dilution of 0.0019 mg/ml. In order to exclude antimicrobial effects of DMSO, DMSO dilution series were tested in a double test. The initial concentration was 20% DMSO and diluted to 0.0004% using the same procedure. As a positive control, 0.1% CHX was tested on each plate in duplicate and diluted down to 0.0002%. Column 11 of each plate contained WCB or MHB and was inoculated as growth control. Column 12 contained only WCB and MHB as blank values for optical comparison. The inoculation of each well up to the column blank value was carried out with 5 µl germ suspension. Only one germ was inoculated per plate (31, 34). Subsequently, the plates with the facultative anaerobic germs and *C. albicans* were incubated at 37°C and 5%–10% CO_2_ atmospheric pressure for 24 h. The anaerobically inoculated plates were also incubated at 37°C for 48 h under anaerobic conditions. Inocula were prepared from one of the growth controls to check the achievement of the desired CFU. In parallel to the inoculum control, smears from the last dilution series were fractionated and incubated in the CO_2_ oven. If aerobic growth became visible the next day, the experiment was discarded.

The turbidity in the wells was assessed under a magnifying lamp. The MIC was defined as the lowest concentration of each active substance at which a visible inhibition of bacterial growth was induced. This means the MIC was visually determined at the concentration at which no turbidity was visible or no growth was visible according to the growth control comparison. If the MIC values in the duplicate extract were different, the higher concentration was evaluated as MIC. If there was more than one concentration level difference, the experiment was repeated. The possibly inhibitory effect of the solvent DMSO was also considered. An extract concentration of 10 mg/ml contains 10% DMSO. Thus, an extract effect could only be considered if the MIC for DMSO was greater than 10% in the same experimental approach. The growth was divided into three strengths, which were recorded in the laboratory protocol with +, ++, +++. The decisive factor is the last well without visible growth. This determines the MIC. The reported values represent the mean values, and the experiments were conducted in duplicate.

### Determination and evaluation of the minimum bactericidal concentration (MBC)

To determine the minimum bactericidal concentration, 10 µl were spread out from each well on a quarter of the culture medium to the dilution at which bacterial growth was clearly visible. In the case of facultatively anaerobic bacteria and *C. albicans*, incubation was performed on CBA plates at 37°C and 5%–10% CO_2_ atmospheric pressure for 24–48 h. The anaerobes were incubated under anaerobic conditions on HCB culture media at 37°C for 4–5 days.

The MBC was defined as a drop in growth of 99.9%. As a guideline, ten colony-forming units per 10 µl smear were allowed. The CFU was determined visually (31). The reported values represent the mean values, and the experiments were conducted in duplicate.

### Determination and evaluation of biofilm formation

The experiments to test the inhibition of biofilm formation were developed after the submission of a paper described in 2014 (35). To test the inhibition of biofilm formation, the clinical isolate R 15-8 of *S. mutans* was used as a biofilm forming, facultative anaerobic bacterium. *S. mutans* R15-8 was isolated from an infected root canal of a tooth that had undergone dental treatment in the Department of Operative Dentistry and Periodontology, University of Freiburg, Germany. The biofilm-forming bacterium *E. faecalis* was used as a control germ. Subcultures were created and incubated at 37°C and 5%–10% CO_2_ atmospheric pressure for 24 h. The next day, the isolates were cultivated in tryptic soy broth (TSB) overnight with the addition of sucrose (Merck KGaA, Darmstadt, Deutschland). The TSB culture medium used includes 2.5 g/L glucose, which facilitates the formation of glucan as a biofilm matrix by *S. mutans*. The CFU of each overnight culture were determined on CBA. The live bacterial count was in a range of 10^8^ CFU/ml. Each well of a 96 well tissue-culture plates (Greiner bio-one, Frickenhausen, Germany) was filled with 180 µl fresh tryptic soy broth (TSB). One extract per plate was tested in a quadruple experiment, again DMSO and CHX dilutions were used as controls. Dilutions from 10 to 0.019 mg/ml were tested. Analogous to the MIC determination CHX from 0.1% to 0.0002% and DMSO from 20% to 0.004%. Afterwards, 20 µl of the overnight culture were pipetted before incubation of the microtiter plates for 24 h at 37°C and 5%–10% CO_2_ atmospheric pressure. After incubation, the liquid was discarded. The plates were washed three times with 200 µl phosphate buffered salt solution (PBS; Life Technologies Inc., Carlsbad, CA, USA) and air dried for 10 min. With 0.1% crystal violet (Carl Roth GmbH + Co KG, Karlsruhe, Deutschland) staining for 10 min was performed. After washing three times with 200 µl distilled water and drying the plates for 10 min at 60°C, the dye was dissolved with 50 µl 99.9% alcohol each. The optical density was determined at 595 nm with a microtiter plate photometer (Tecan Group AG, Männedorf, Switzerland).

Three categories were formed on the basis of thresholds. The first threshold value was formed by adding three times the value of the standard deviation of the negative control to the actual measured value of the negative control. The second threshold was defined as three times the value of the first threshold. Values below the first threshold value were used to inhibit biofilm formation. Values higher than the first threshold value, but lower as the second, were considered moderate biofilm formation. Values above the second threshold were considered as strong biofilm formation. This approach was utilized to eliminate false positive results for biofilm formation.

### Statistical analysis

For analysis of the biofilm plate assay, *T*-tests were applied between the logarithmic adsorption values (basis 10) of the extracts and the two control groups, respectively, with a Bonferroni-correction due to multiple testing. For graphical presentation of the results scatter plots were used. All computations were done with STATA (Version 17.0, College Station, TX, USA).

## Results

All extracts, except the hydroalcoholic extract of *V. myrtillus*, showed an antibacterial effect at concentrations ranging from 10 to 0.15 mg/ml. The most significant and consistent results were generally obtained with anaerobic bacteria. However, inhibition of the yeast was challenging or minimal. Overall, The extracts of *L. stoechas*, *S. thymbra*, *S. parnassica*, and the hydroalcoholic extract of *V. myrtillus* yielded an antibiofilm effect at concentrations up to 1.25 mg/ml.

### HPTLC analysis

To obtain the chemical profile of the plant extracts, a rapid and accurate analytical method was developed using HPTLC (High-Performance Thin Layer Chromatography). Visualization of the plates at 254 and 366 nm revealed that the extracts possessed a diverse chemical content, and major active compound categories were detected. The analysis primarily indicated the presence of phenolic compounds. Rosmarinic acid was identified as the main compound in *Mentha*, *Lavandula*, *Satureja*, *Phlomis*, and *Stachys*. Phenylethanol glycosides, such as acteoside, and flavonoid glucosides of hypolaetin, methylhypolaetin, isoscutellarein, and methylisoscutellarein, were found to be the main compounds in *Sideritis* species. A. *taygetea* extracts were rich in flavonoids, specifically derivatives of apigenin and luteolin. *V. myrtillus* extracts were abundant in anthocyanins, particularly cyanidin-3-glucoside and delphinidin-3-glucoside.

### Sideritis euboea and Satureja thymbra

*Sideritis euboea* has demonstrated effectiveness against all anaerobic bacteria, as well as against *S. aureus* and *S. oralis*, exhibiting inhibitory and bactericidal effects at dilutions of 1.25 mg/ml, as indicated in Table 1. The most notable results were observed against *P. gingivalis* and *P. micra*, with minimum inhibitory concentration (MIC) and minimum bactericidal concentration (MBC) values of 0.3 mg/ml each.

**Table 1:** Antimicrobial activity in mg ml^−1^ of *Sideritis euboea* hydroalcoholic extract.

*Sideritis euboea*				
Sample	Hydroalcoholic extract		DMSO (%)	
(in mg ml^−1^)	MIC	MBC	MIC	MBC
*Streptococcus mutans* DSM 20523	2.5	10	10	>20
*Streptococcus sobrinus* DSM 20381	2.5	10	20	>20
*Streptococcus oralis* ATCC 35037	1.25	1.25	10	20
*Enterococcus faecalis* ATCC 29212	2.5	10	20	>20
*Candida albicans* DSM 1386	10	10	10	>20
*Escherichia coli* ATCC 25922	10	10	20	>20
*Staphylococcus aureus* ATCC 25923	1.25	1.25	20	>20
*Porphyromonas gingivalis* W381	0.3	0.3	20	20
*Prevotella intermedia* MSP 34	0.6	0.6	2.5	2.5–5
*Fusobacterium nucleatum* ATCC 25586	1.25	1.25	10	10
*Parvimonas micra* ATCC 23195	0.3	0.3	2.5	20

MIC, extract concentration at which the OD measurement revealed minimal bacterial growth. MBC, extract concentration at which a 3-Log reduction (99.9%) of the bacterial growth was induced.

*Sideritis syriaca* (Table 2) demonstrated effective inhibition against *S. mutans*, with a minimum inhibitory concentration (MIC) of 1.25 mg/ml. Additionally, *S. aureus*, *P. gingivalis*, and *P. micra* displayed MIC values of 0.6 mg/ml or below, and except for *S. oralis*, minimum bactericidal concentration (MBC) values within the same range. However, for *P. intermedia*, clear results could not be obtained as the MIC/MBC values of the extract and DMSO were too close to each other. The same was observed for *E. coli* and *C. albicans*.

**Table 2:** Antimicrobial activity in mg ml^−1^ of *Sideritis syriaca* hydroalcoholic extract.

*Sideritis syriaca*				
Sample	Hydroalcoholic extract		DMSO (%)	
(in mg ml^−1^)	MIC	MBC	MIC	MBC
*Streptococcus mutans* DSM 20523	1.25	5	5	>20
*Streptococcus sobrinus* DSM 20381	2.5	10	20	20
*Streptococcus oralis* ATCC 35037	0.6	2.5	10	20
*Enterococcus faecalis* ATCC 29212	5	10	20	>20
*Candida albicans* DSM 1386	10	10	10	20
*Escherichia coli* ATCC 25922	5	10	10	20
*Staphylococcus aureus* ATCC 25923	0.3	0.6	10	>20
*Porphyromonas gingivalis* W381	0.3	0.3	20	20
*Prevotella intermedia* MSP 34	1.25	1.25	2.5	2.5
*Fusobacterium nucleatum* ATCC 25586	2.5	5	10	10
*Parvimonas micra* ATCC 23195	0.3	0.6	5	20

MIC, extract concentration at which the OD measurement revealed minimal bacterial growth. MBC, extract concentration at which a 3-Log reduction (99.9%) of the bacterial growth was induced.

### Mentha longifolia and Mentha aquatica

For *Mentha longifolia* (Table 3), inhibitory effects were observed at a concentration of 2.5 mg/ml against *S. mutans*, *S. sobrinus*, and *S. aureus*. Notably, *P. gingivalis* showed a minimum inhibitory concentration (MIC) of 0.3 mg/ml and a minimum bactericidal concentration (MBC) of 0.6 mg/ml, while *P. micra* exhibited an MIC of 0.6 mg/ml and an MBC of 0.6 mg/ml, indicating susceptibility to lower concentrations as is typical for anaerobic bacteria. However, for *P. intermedia*, the MIC and MBC values were indistinguishable from those of the DMSO control.

**Table 3:** Antimicrobial activity in mg ml^−1^ of *Mentha longifolia* hydroalcoholic extract.

*Mentha longifolia*				
Sample	Hydroalcoholic extract		DMSO (%)	
(in mg ml^−1^)	MIC	MBC	MIC	MBC
*Streptococcus mutans* DSM 20523	2.5	10	10	>20
*Streptococcus sobrinus* DSM 20381	2.5	10	20	>20
*Streptococcus oralis* ATCC 35037	1.25	1.25	10	20
*Enterococcus faecalis* ATCC 29212	5	>10	20	>20
*Candida albicans* DSM 1386	5	10	10	20
*Escherichia coli* ATCC 25922	5	10	20	20
*Staphylococcus aureus* ATCC 25923	2.5	2.5	20	>20
*Porphyromonas gingivalis* W381	0.3	0.6	10	10
*Prevotella intermedia* MSP 34	1.25	5	2.5	2.5
*Fusobacterium nucleatum* ATCC 25586	2.5	2.5	10	10
*Parvimonas micra* ATCC 23195	0.6	0.6	5	20

MIC, extract concentration at which the OD measurement revealed minimal bacterial growth. MBC, extract concentration at which a 3-Log reduction (99.9%) of the bacterial growth was induced.

*Mentha aquatica* (Table 4) exhibited notable results in relation to *E. faecalis*. The extract demonstrated inhibition of the bacteria at a concentration of 0.3 mg/ml, although the minimum bactericidal concentration (MBC) was found to be at a concentration of 10 mg/ml. However, no significant results were obtained against the tested *Streptococcus* species. The values for *P. intermedia*, *F. nucleatum*, and *P. micra* were also similar to those of the DMSO controls, making them inconclusive. As for *C. albicans* and *E. coli*, the observed effects can be attributed to the DMSO rather than the extract, suggesting no direct impact of the extract on these microorganisms.

**Table 4:** Antimicrobial activity in mg ml^−1^ of *Mentha aquatica* hydroalcoholic extract.

*Mentha aquatica*				
Sample	Hydroalcoholic extract		DMSO (%)	
(in mg ml^−1^)	MIC	MBC	MIC	MBC
*Streptococcus mutans* DSM 20523	5	10	5	>20
*Streptococcus sobrinus* DSM 20381	5	10	20	20
*Streptococcus oralis* ATCC 35037	5	10	10	20
*Enterococcus faecalis* ATCC 29212	0.3	10	20	>20
*Candida albicans* DSM 1386	10	10	10	20
*Escherichia coli* ATCC 25922	10	10	10	20
*Staphylococcus aureus* ATCC 25923	0.6	2.5	10	>20
*Porphyromonas gingivalis* W381	0.6	2.5	20	20
*Prevotella intermedia* MSP 34	1.25	5	2.5	2.5
*Fusobacterium nucleatum* ATCC 25586	5	10	10	10
*Parvimonas micra* ATCC 23195	2.5	5	5	20

MIC, extract concentration at which the OD measurement revealed minimal bacterial growth. MBC, extract concentration at which a 3-Log reduction (99.9%) of the bacterial growth was induced.

### Satureja thymbra and Satureja parnassica

*Satureja thymbra* (Table 5) yielded favorable results in inhibiting anaerobic bacteria and *S. oralis*. However, it is worth noting that the possibility of a DMSO effect cannot be completely ruled out when testing against *F. nucleatum*. *E. faecalis* also demonstrated sensitivity to the extract, with a minimum inhibitory concentration (MIC) of 1.25 mg/ml.

**Table 5:** Antimicrobial activity in mg ml^−1^ of *Satureja thymbra* hydroalcoholic extract.

*Satureja thymbra*				
Sample	Hydroalcoholic extract		DMSO (%)	
(in mg ml^−1^)	MIC	MBC	MIC	MBC
*Streptococcus mutans* DSM 20523	5	5	10	>20
*Streptococcus sobrinus* DSM 20381	2.5	5	20	>20
*Streptococcus oralis* ATCC 35037	1.25	2.5	10	20
*Enterococcus faecalis* ATCC 29212	1.25	5	20	>20
*Candida albicans* DSM 1386	10	10	20	>20
*Escherichia coli* ATCC 25922	5	10	20	20
*Staphylococcus aureus* ATCC 25923	0.6	1.25	20	>20
*Porphyromonas gingivalis* W381	0.6	0.6	20	20
*Prevotella intermedia* MSP 34	1.25	1.25	1.25	2.5
*Fusobacterium nucleatum* ATCC 25586	1.25	1.25	5	10
*Parvimonas micra* ATCC 23195	0.6	1.25	5	10

MIC, extract concentration at which the OD measurement revealed minimal bacterial growth. MBC, extract concentration at which a 3-Log reduction (99.9%) of the bacterial growth was induced.

*Satureja parnassica* showed a limited impact on *S. mutans*, as indicated in Table 6. However, for *S. oralis*, a minimum inhibitory concentration (MIC) of 2.5 mg/ml and a minimum bactericidal concentration (MBC) of 5 mg/ml were determined. Similar to *Mentha aquatica*, *S. parnassica* exhibited a low MIC (0.6 mg/ml) against *E. faecalis*, although the MBC was significantly higher at 10 mg/ml. *S. aureus*, *P. gingivalis*, and *P. micra* displayed sensitivity to the extract's effects.

**Table 6:** Antimicrobial activity in mg ml^−1^ of *Satureja parnassica* hydroalcoholic extract.

*Satureja parnassica*				
Sample	Hydroalcoholic extract		DMSO (%)	
(in mg ml^−1^)	MIC	MBC	MIC	MBC
*Streptococcus mutans* DSM 20523	5	10	10	>20
*Streptococcus sobrinus* DSM 20381	5	10	20	>20
*Streptococcus oralis* ATCC 35037	2.5	5	10	20
*Enterococcus faecalis* ATCC 29212	0.6	10	20	>20
*Candida albicans* DSM 1386	10	10	20	>20
*Escherichia coli* ATCC 25922	2.5	10	20	20
*Staphylococcus aureus* ATCC 25923	0.6	2.5	20	>20
*Porphyromonas gingivalis* W381	0.6	1.25	20	20
*Prevotella intermedia* MSP 34	1.25	1.25	1.25	2.5
*Fusobacterium nucleatum* ATCC 25586	2.5	2.5	5	10
*Parvimonas micra* ATCC 23195	1.25	1.25	5	10

MIC, extract concentration at which the OD measurement revealed minimal bacterial growth. MBC, extract concentration at which a 3-Log reduction (99.9%) of the bacterial growth was induced.

### Stachys spinosa and Achillea taygetea

Table 7 provides a summary of the results for the *Stachys spinosa* extract. Similar to the hydroalcoholic extract of *Satureja thymbra*, excellent values were observed between 0.3 and 1.25 mg/ml for *P. gingivalis*, *F. nucleatum*, and *P. micra*. However, when testing against *P. intermedia*, the possibility of a DMSO effect could not be excluded. A significant effect against *S. oralis* was evident. However, weak minimum inhibitory concentration (MIC) values were obtained for the other tested microorganisms, and these values could not be confirmed by the minimum bactericidal concentration (MBC).

**Table 7:** Antimicrobial activity in mg ml^−1^ of *Stachys spinosa* hydroalcoholic extract.

*Stachys spinosa*				
Sample	Hydroalcoholic extract		DMSO (%)	
(in mg ml^−1^)	MIC	MBC	MIC	MBC
*Streptococcus mutans* DSM 20523	2.5	>10	10	>20
*Streptococcus sobrinus* DSM 20381	5	10	10	>20
*Streptococcus oralis* ATCC 35037	1.25	2.5	10	20
*Enterococcus faecalis* ATCC 29212	10	>10	20	>20
*Candida albicans* DSM 1386	5	>10	10	20
*Escherichia coli* ATCC 25922	10	>10	20	>20
*Staphylococcus aureus* ATCC 25923	5	10	20	>20
*Porphyromonas gingivalis* W381	0.3	0.6	10	10
*Prevotella intermedia* MSP 34	2.5	2.5	1.25	2.5
*Fusobacterium nucleatum* ATCC 25586	0.6	1.25	5	5
*Parvimonas micra* ATCC 23195	0.3	0.6	2.5	10

MIC, extract concentration at which the OD measurement revealed minimal bacterial growth. MBC, extract concentration at which a 3-Log reduction (99.9%) of the bacterial growth was induced.

The extract of *Achillea taygetea* exhibited similar effects, as shown in Table 8. Good minimum inhibitory concentration (MIC) values ranging from 1.25 mg/ml and minimum bactericidal concentration (MBC) values below 2.5 mg/ml were achieved against *S. oralis*, *P. gingivalis*, and *F. nucleatum*. For *P. micra*, both MIC and MBC were even observed at a concentration of 0.15 mg/ml, indicating strong inhibitory and bactericidal effects. However, no clear extract effect could be demonstrated against *S. mutans*, *S. sobrinus*, *E. faecalis*, *E. coli*, and *P. intermedia*. Furthermore, the extract did not exhibit significant inhibition against *C. albicans*.

**Table 8:** Antimicrobial activity in mg ml^−1^ of *Achillea taygetea* hydroalcoholic extract.

*Achillea taygetea*				
Sample	Hydroalcoholic extract		DMSO (%)	
(in mg ml^−1^)	MIC	MBC	MIC	MBC
*Streptococcus mutans* DSM 20523	5	>10	10	>20
*Streptococcus sobrinus* DSM 20381	5	>10	10	>20
*Streptococcus oralis* ATCC 35037	1.25	2.5	10	20
*Enterococcus faecalis* ATCC 29212	10	>10	20	>20
*Candida albicans* DSM 1386	10	10	10	20
*Escherichia coli* ATCC 25922	10	>10	20	>20
*Staphylococcus aureus* ATCC 25923	5	10	20	>20
*Porphyromonas gingivalis* W381	0.3	0.3	10	10
*Prevotella intermedia* MSP 34	2.5	2.5	1.25	2.5
*Fusobacterium nucleatum* ATCC 25586	0.6	0.6	5	5
*Parvimonas micra* ATCC 23195	0.15	0.15	2.5	10

MIC, extract concentration at which the OD measurement revealed minimal bacterial growth. MBC, extract concentration at which a 3-Log reduction (99.9%) of the bacterial growth was induced.

### Phlomis cretica

*Phlomis cretica* (Table 9) demonstrated reliable activity against the anaerobic bacteria *P. gingivalis*, *F. nucleatum*, and *P. micra*, as well as the facultative anaerobic bacterium *S. oralis*. Notably, the extract exhibited excellent MIC and MBC values of 0.6 mg/ml for *S. mutans*, underscoring its effectiveness against this particular microorganism.

**Table 9:** Antimicrobial activity in mg ml^−1^ of *Phlomis cretica* hydroalcoholic extract.

*Phlomis cretica*				
Sample	Hydroalcoholic extract		DMSO (%)	
(in mg ml^−1^)	MIC	MBC	MIC	MBC
*Streptococcus mutans* DSM 20523	0.6	0.6	10	>20
*Streptococcus sobrinus* DSM 20381	5	10	20	20
*Streptococcus oralis* ATCC 35037	1.25	1.25	10	20
*Enterococcus faecalis* ATCC 29212	5	10	20	>20
*Candida albicans* DSM 1386	10	10	10	>20
*Escherichia coli* ATCC 25922	10	10	20	>20
*Staphylococcus aureus* ATCC 25923	5	5	20	>20
*Porphyromonas gingivalis* W381	0.6	0.6	20	20
*Prevotella intermedia* MSP 34	1.25	1.25	2.5	5
*Fusobacterium nucleatum* ATCC 25586	1.25	1.25	10	10
*Parvimonas micra* ATCC 23195	0.15	0.15	2.5	20

MIC, extract concentration at which the OD measurement revealed minimal bacterial growth. MBC, extract concentration at which a 3-Log reduction (99.9%) of the bacterial growth was induced.

### Lavandula stoechas

The testing of *Lavandula stoechas* (Table 10) revealed minimum inhibitory concentration (MIC) concentrations of 2.5 mg/ml for *S. mutans*, *S. sobrinus*, and *S. oralis*. However, the minimum bactericidal concentration (MBC) for *S. mutans* and *S. sobrinus* was relatively high at 10 mg/ml compared to the MIC value. *P. gingivalis* could be inhibited at a very low concentration of 0.15 mg/ml, and at twice that concentration, it was effectively killed. *S. aureus* (MIC = 1.25 mg/ml; MBC = 2.5 mg/ml) and *P. micra* (MIC and MBC = 0.6 mg/ml) demonstrated the expected sensitivity to the extract.

**Table 10:** Antimicrobial activity in mg ml^−1^ of *Lavandula stoechas* hydroalcoholic extract.

*Lavandula stoechas*				
Sample	Hydroalcoholic extract		DMSO (%)	
(in mg ml^−1^)	MIC	MBC	MIC	MBC
*Streptococcus mutans* DSM 20523	2.5	>10	10	>20
*Streptococcus sobrinus* DSM 20381	2.5	10	20	>20
*Streptococcus oralis* ATCC 35037	2.5	2.5	10	20
*Enterococcus faecalis* ATCC 29212	5	10	20	>20
*Candida albicans* DSM 1386	10	10	10	20
*Escherichia coli* ATCC 25922	5	10	20	20
*Staphylococcus aureus* ATCC 25923	1.25	2.5	20	>20
*Porphyromonas gingivalis* W381	0.15	0.3	10	10
*Prevotella intermedia* MSP 34	1.25	2.5	2.5	2.5
*Fusobacterium nucleatum* ATCC 25586	5	>10	10	10
*Parvimonas micra* ATCC 23195	0.6	0.6	5	20

MIC, extract concentration at which the OD measurement revealed minimal bacterial growth. MBC, extract concentration at which a 3-Log reduction (99.9%) of the bacterial growth was induced.

### Vaccinium myrtillus

The hydroalcoholic extract of *Vaccinium myrtillus* (Table 11) exhibited minimal notable effects. The measured inhibitory concentration of 0.6 mg/ml for *P. micra* is likely not significant, as it aligns closely with the effects of DMSO, particularly considering the MBC of 10 mg/ml and the MBC of 20 mg/ml.

**Table 11:** Antimicrobial activity in mg ml^−1^ of *Vaccinium myrtillus* hydroalcoholic extract.

*Vaccinium myrtillus*				
Sample	Hydroalcoholic extract		DMSO (%)	
(in mg ml^−1^)	MIC	MBC	MIC	MBC
*Streptococcus mutans* DSM 20523	10	>10	5	>20
*Streptococcus sobrinus* DSM 20381	na	>10	20	>20
*Streptococcus oralis* ATCC 35037	10	10	10	20
*Enterococcus faecalis* ATCC 29212	na	>10	20	>20
*Candida albicans* DSM 1386	10	>10	10	20
*Escherichia coli* ATCC 25922	10	>10	20	>20
*Staphylococcus aureus* ATCC 25923	10	>10	20	>20
*Porphyromonas gingivalis* W381	5	>10	20	20
*Prevotella intermedia* MSP 34	2.5	5	2.5	2.5
*Fusobacterium nucleatum* ATCC 25586	5	10	10	10
*Parvimonas micra* ATCC 23195	0.6	10	5	20

MIC, extract concentration at which the OD measurement revealed minimal bacterial growth. MBC, extract concentration at which a 3-Log reduction (99.9%) of the bacterial growth was induced. na, No activity observed, MIC or MBC of extracts and DMSO at 10.00 mg ml^−1^ and 20%, respectively.

Key findings are summarized in Table 12.

**Table 12:** Overview of the lowest MIC/MBC values against the tested pathogens.

Extracts	Lowest MIC/pathogens	Lowest MBC/pathogens
*Mentha aquatica*	0.3 mg/ml/*E. faecalis*	2.5 mg/ml/*P. gingivalis*, *S. aureus*
*Mentha longifolia*	0.3 mg/ml/*P. gingivalis*	0.6 mg/ml/*P. gingivalis*, *P. micra*
*Sideritis euboea*	0.3 mg/ml/*P. gingivalis*, *P. micra*	0.3 mg/ml/*P. gingivalis*, *P. micra*
*Sideritis syriaca*	0.3 mg/ml/*P. gingivalis*, *P. micra*, *S. aureus*	0.3 mg/ml/*P. gingivalis*
*Stachys spinosa*	0.3 mg/ml/*P. gingivalis*, *P. micra*	0.6 mg/ml/*P. gingivalis*, *P. micra*
*Satureja parnassica*	0.6 mg/ml/*P. gingivalis*, *E. faecalis*, *S. aureus*	1.25 mg/ml/*P. gingivalis*, *P. micra*, *P. intermedia*
*Satureja thymbra*	0.6 mg/ml/*P. gingivalis*, *P. micra*, *S. aureus*	0.6 mg/ml/*P. gingivalis*
*Lavandula stoechas*	0.15 mg/ml/*P. gingivalis*	0.3 mg/ml/*P. gingivalis*
*Achillea taygetea*	0.15 mg/ml/*P. micra*	0.15 mg/ml/*P. micra*
*Phlomis cretica*	0.15 mg/ml/*P. micra*	0.15 mg/ml/*P. micra*
*Vaccinium myrtillus*	0.6 mg/ml/*P. micra*	5 mg/ml/*P. intermedia*

### Results of the biofilm plate assay

**Figure 1:**
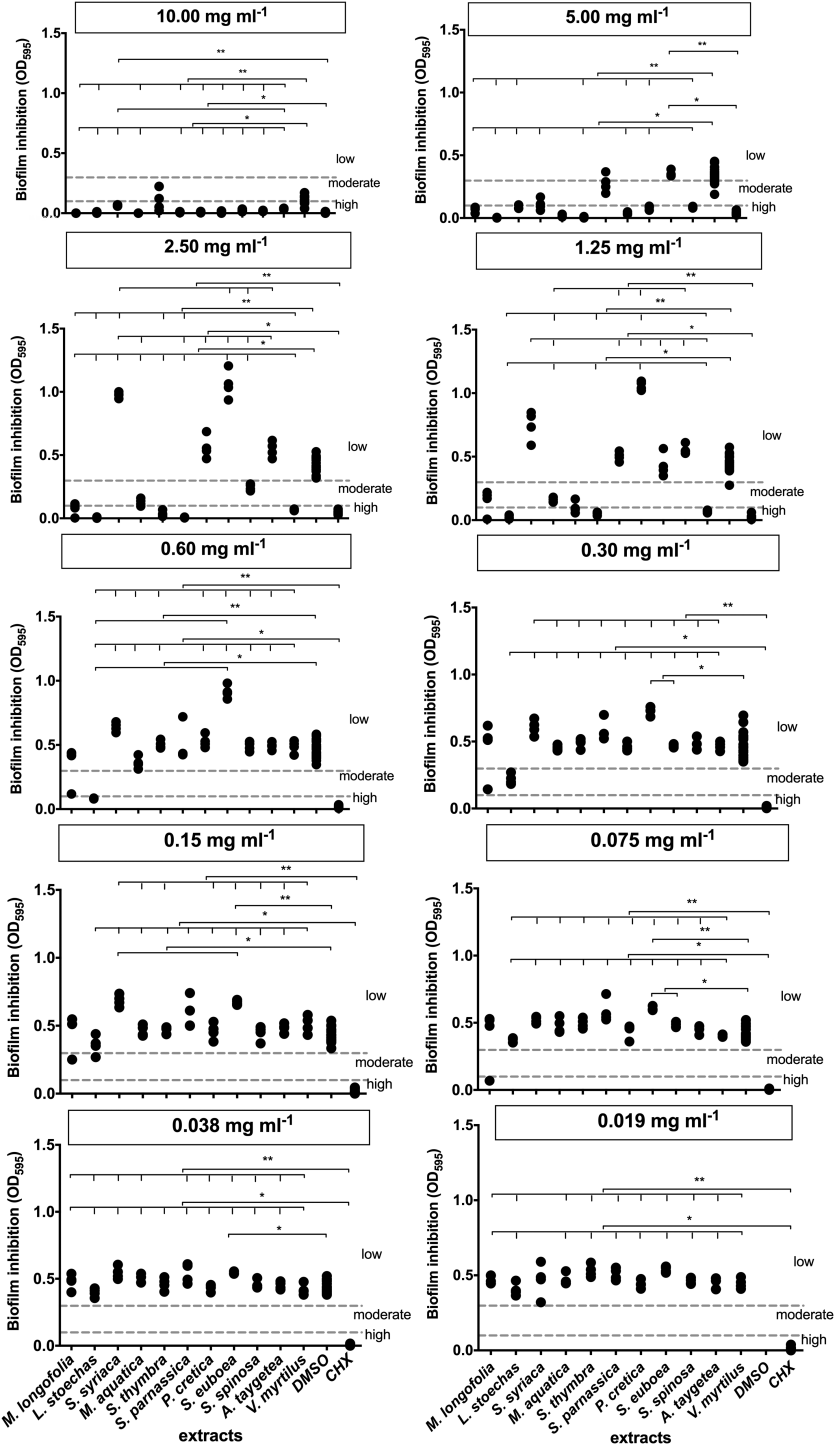
Results of the biofilm inhibition test. **Significance less then 1%, *Significance less then 5%.

The extracted values were compared to the controls of DMSO and CHX, and the significance was evaluated (Figure 1). The significance level of less than 1% or less than 5% is depicted in the diagrams. At a concentration of 10 mg/ml, all extracts, except *S. thymbra*, exhibited high biofilm inhibition (*p* ≤ 0.024). DMSO already showed scattered but moderate inhibition at high concentrations. At a lower concentration of 5 mg/ml, *A. taygetea* extract demonstrated significantly increased values of biofilm formation (*p* = 1). *P. cretica* fell into the category of mild biofilm inhibitors at this concentration (*p* = 1). Extracts of *M. longifolia* (*p* = 0.007), *L. stoechas* (*p* = 0.001), *S. thymbra* (*p* = 0.001), *S. parnassica* (*p* = 0.001), and *V. myrtillus* (*p* = 0.001) exhibited high levels of biofilm inhibition at 2.50 mg/ml.

Furthermore, extracts of *L. stoechas* (*p* = 0.001), *S. parnassica* (*p* = 0.0001), and *V. myrtillus* (*p* = 0.0001) showed activity at 1.25 mg/ml. The optical density (OD) of *S. euboea* (*p* = 0.0001) was notably above the second threshold value. Only *L. stoechas* (*p* = 0.001) achieved biofilm formation inhibition at 0.60 mg/ml. *M. longifolia* (*p* = 1) exhibited a measurement in the moderate range, which should be considered as an outlier. From a concentration of 0.015 mg/ml, *L. stoechas* (*p* = 1) reached the threshold for mild biofilm inhibition but lost effectiveness at 0.075 mg/ml. *M. longifolia* again exhibited an outlier without evaluation.

None of the tested extracts produced moderate biofilm inhibition at a concentration of 0.038 mg/ml. Consequently, at an even lower concentration of 0.019 mg/ml, very weak or no inhibition was observed.

## Discussion

The selected plants are commonly found in the Mediterranean region. *M. longifolia*, *L. stoechas*, *S. syriaca*, *M. aquatica*, *S. thymbra*, *S. parnassica*, *P. cretica*, *S. euboea*, and *S. spinosa* belong to the Lamiaceae family, while *A. taygetea* belongs to the Asteraceae family, and *V. myrtillus* belongs to the Ericaceae family. Testing a greater variety of oral bacteria would yield more comprehensive information regarding the antimicrobial activity of the extracts. However, in our manuscript, we included both Gram-negative and Gram-positive bacteria because the cell envelope is crucial for the mechanisms of all antimicrobial compounds in general. Additionally, we also included the most common oral fungus, *Candida albicans*.

The tested oral streptococcal strains, frequently isolated from the supragingival oral biofilm, have been associated with dental caries according to the ecological plaque hypothesis (36). Conversely, the tested anaerobic oral bacteria (*F. nucleatum*, *P. gingivalis*, *P. intermedia*, and *Parvimonas micra*) have been frequently isolated from the subgingival oral biofilm, which is associated with periimplantitis and periodontitis (37–39). *Candida albicans*, frequently isolated from the supragingival oral biofilm, has been associated with early childhood caries (40, 41). *Enterococcus faecalis* has been described as one of the major infectious bacteria in endodontic infections (42).

An analysis of tea derived from *S. syriaca* demonstrated potent antimicrobial activity against *S. aureus*, indicating strong inhibitory effects. Additionally, the tea exhibited moderate inhibition of *E. coli* and *E. faecalis*, suggesting a moderate impact on these bacterial strains (43). The significant effect of *S. syriaca* on *S. aureus* was further supported by this experiment, with a minimum inhibitory concentration (MIC) of 0.3 mg/ml and a minimum bactericidal concentration (MBC) of 0.6 mg/ml. In a previous study conducted in 2001, *S. syriaca* was also attributed with high antimicrobial activity. This effect was mainly attributed to the presence of carvacrol, which was found in substantial amounts in the essential oil of *S. syriaca* (44). Among other things, carvacrol is utilized in other experiments as a rationale for its antimicrobial effect against microorganisms (45).

In a previous study, both *M. aquatica* and *M. longifolia* demonstrated a significant antimicrobial effect, which is consistent with the findings of the current work (46). Furthermore, the antimicrobial activity of a hydroalcoholic extract of *M. longifolia* against *S. aureus* and *E. coli* was reported at concentrations of 1, 3, and 5 mg/ml (47). These values align closely with the minimum inhibitory concentrations (MIC) determined in this study, which were 5 mg/ml for *E. coli* and 2.5 mg/ml for *S. aureus*.

The essential oils of the two *Satureja* species have undergone multiple testing in various studies. In a study conducted in 2006, essential oils were extracted from *S. parnassica* and *S. thymbra*, and their compositions were differentiated based on the time of harvest. Carvacrol and thymol were identified as the primary isomers in each oil produced. Carvacrol was found to be the predominant component during the flowering period, while thymol became more abundant shortly before and after flowering. Additionally, the levels of precursor compounds of these substances increased over time. The oils obtained from the flowering plants exhibited the lowest minimum inhibitory concentration (MIC) values. Furthermore, the study confirmed the inherently stronger effect of these oils on Gram-positive pathogens compared to Gram-negative ones, as mentioned in the above cited research (48).

*S. spinosa* exhibited a notable effect on Gram-negative anaerobic bacteria in the experiments. It also achieved moderate biofilm inhibition at a concentration of 2.5 mg/ml. There are currently no other antimicrobial investigations of these plant substances available for comparison with the obtained results. In a chemical analysis of the above-ground parts of the plant as a hydroalcoholic extract, stachyspinosides, belonging to the flavonoid group, as well as three secondary plant substances in the form of iridoids, were detected (49).

*A. taygetea* demonstrated an effect on the Gram-positive pathogen *S. oralis* (MIC = 1.25 mg/ml; MBC = 2.5 mg/ml), but not on the other streptococci or *E. faecalis*. Additionally, no significant effect was observed against the tested fungus. However, the Gram-negative anaerobes and *P. micra* exhibited greater sensitivity to the extract.

Chemical analysis of the extract of *A. taygetea* revealed the presence of α- and β-pinene, camphene, 1,8-cineole, camphor, and α-terpineol in significant proportions compared to other *Achillea* species (50). The demonstrated antimicrobial effect of *A. taygetea* is attributed to 1,8-cineole and camphor based on individual tests of these components (50, 51).

There is currently no available literature on the antimicrobial activity of *P. cretica* specifically, but other *Phlomi*s species have been studied. For example, the essential oil of *Phlomis lantana* was found to contain significant amounts of α-pinene, limonene, and trans-caryophyllene. This oil was tested for its minimal inhibitory concentration against Gram-negative and Gram-positive bacteria, as well as fungi including *C. albicans*. It exhibited a stronger effect against Gram-negative pathogens like *P. aeruginosa* and *E. faecalis* compared to Gram-positive ones. Further testing of the three mentioned chemical components for their minimal inhibitory concentration against microorganisms revealed that the activity of the oil was likely attributed to α-pinene, while limonene was found to be completely inactive (52). In the present study, the most notable effect of *P. cretica* was observed on the Gram-positive bacterium *P. micra*. Other tested Gram-negative bacteria also showed sensitivity, except for *E. coli*, which required a higher concentration (MIC and MBC at 10 mg/ml). Additionally, the Gram-positive streptococci *S. mutans* and *S. oralis* were significantly affected, while the inhibition of biofilm formation by *P. cretica* was only observed at higher concentrations.

In this study, *L. stoechas* demonstrated moderate biofilm inhibition at low concentrations of 0.30 mg/ml. However, in another study by Gursoy et al., an essential oil of *L. stoechas* was not included in the biofilm experiments due to its high minimum inhibitory concentration (MIC) values (53). Chemical analysis conducted in the study by Gursoy et al. identified camphor, fenchone, and 1,8-cineole (also known as eucalyptol) as the main components of the essential oil (53–55). In the literature, fenchone, one of the main components, has been evaluated as weakly antimicrobial, which may explain the limited antimicrobial behavior of *L. stoechas* essential oil observed in Dadalioglu's study. However, in the experiments described here, *L. stoechas* extract exhibited an effect on the tested anaerobic bacteria as well as on *S. aureus* and *S. oralis*.

*V. myrtillus* (blueberry) has been extensively studied for its antioxidant and hypoglycemic effects in existing research. A qualitative analysis of blueberry stems, leaves, and fruits has shown that all components of the plant are suitable sources of phenolic compounds (56). In this study, the hydroalcoholic extract of blueberry exhibited an effect on *P. micra* with a minimum inhibitory concentration (MIC) of 0.6 mg/ml, but this effect was diminished by a minimum bactericidal concentration (MBC) of 10 mg/ml. Regarding the inhibition of biofilm formation, the extract demonstrated a significant inhibition at concentrations up to 1.25 mg/ml, but at lower concentrations, it was just above the threshold for low inhibitors. This observation is consistent with a review that described the inhibition of biofilm formation by blueberry against *S. mutans* as moderate when compared to its related fruit, cranberry (57). Recent studies have examined *Vaccinium myrtillus* (blueberry) for its antioxidant, hypoglycemic, and antimicrobial properties (58, 59). The hydroalcoholic extract showed an inhibitory effect on *P. micra*, with a minimum inhibitory concentration (MIC) of 0.6 mg/ml, but the bactericidal activity required a much higher concentration of 10 mg/ml, indicating a weak effect. The extract also inhibited biofilm formation at concentrations up to 1.25 mg/ml, though lower concentrations had marginal effects, consistent with previous findings on Streptococcus mutans. Overall, while V. myrtillus has antimicrobial activity, its effectiveness in biofilm inhibition and bactericidal action is less pronounced compared to related species like *Vaccinium macrocarpon* (cranberry), warranting further research into its active compounds and potential synergies (60–62).

Herbal products have been extensively studied for their antimicrobial properties due to their diverse bioactive compounds. Previous research has demonstrated the antimicrobial effects of *Satureja thymbra* and *Satureja parnassica*, with essential oils rich in carvacrol and thymol showing strong inhibitory activity against both Gram-positive and Gram-negative bacteria, particularly *Staphylococcus aureus* and *Escherichia coli* (63). Similarly, *Mentha longifolia* and *Mentha aquatica* have exhibited significant antimicrobial activity in multiple studies, with hydroalcoholic extracts displaying low minimum inhibitory concentration (MIC) values against *S. aureus* and *E. coli* (30, 64). This activity is attributed to their high concentrations of rosmarinic acid and menthol. *Vaccinium myrtillus* (blueberry) has also been investigated for its phenolic compounds, which effectively inhibit biofilm formation by *Streptococcus mutans* and related oral pathogens, exhibiting comparable activity to that of cranberry (65). Additionally, *L. stoechas*, which contains camphor, fenchone, and 1,8-cineole, has shown moderate antimicrobial effects, particularly against anaerobic bacteria and fungi, although previous studies reported variability in biofilm inhibition depending on the extraction methods used (66, 67). Other Mediterranean plants, such as *Phlomis lantana* and *Sideritis syriaca*, have demonstrated antimicrobial properties against both oral and systemic pathogens (68, 69). These effects are primarily attributed to their diterpenoids, flavonoids, and phenolic compounds. The antimicrobial and antibiofilm properties of these plants align with the ecological plaque hypothesis, highlighting their potential role in managing oral diseases caused by pathogenic biofilms. Despite these promising findings, more comprehensive studies are required to confirm their efficacy, particularly *in vivo*, and to evaluate their safety and potential for incorporation into oral care products.

This study offers valuable insights, but it has several limitations. It was conducted *in vitro*, which does not fully replicate the complexities of the oral environment, such as the presence of saliva and host-microbe interactions. Furthermore, only a limited range of oral microorganisms were tested, omitting many that are relevant to biofilm complexity. There may also be challenges in achieving effective concentrations of the plant extracts in clinical applications. The exact mechanisms of action, as well as any potential synergistic or antagonistic effects of the compounds within the extracts, remain unclear. Additionally, the study did not evaluate cytotoxicity on human tissues nor did it include comparisons with other oral hygiene products. Future research should address these gaps through *in vivo* studies, broader microbial testing, and safety evaluations. Potential biases in extraction methods could arise from solvent effects, such as the use of DMSO, which may affect microbial growth at higher concentrations. To ensure repeatability, MIC/MBC and biofilm assays were performed in duplicate with standardized protocols, including appropriate controls and statistical analysis, to minimize variability and confirm consistent results.

All tested extracts exhibited antimicrobial effects. However, none of the extracts were effective at concentrations as low as 0.15 mg/ml, and most of them showed effectiveness at concentrations ranging from 2.5 to 5 mg/ml. This can present challenges in terms of procurement, production, and dosage forms. Consequently, it seems logical to attribute the observed effects to specific ingredients or the interaction of multiple ingredients. However, comprehensive analytical methods beyond HPTLC analysis are needed to elucidate and understand the precise mechanisms of action. It is recommended that further studies investigate the hypothesis attributing the antimicrobial and antibiofilm effects to specific pure substances within the tested extracts. Additional tests on the toxicity of the plant extracts introduced in our study are also required. In the *in vitro* experiments conducted in our study, chlorhexidine digluconate (CHX) served as a positive control. To further evaluate the effects of mouthwashes containing CHX compared to the tested plant extracts, future studies should be conducted *in vivo* (70, 71).

The antimicrobial properties of Mediterranean plant extracts present promising applications in oral healthcare products like mouthwashes and toothpaste. These extracts can inhibit oral pathogens, particularly anaerobic bacteria and biofilm formation, making them potential natural alternatives to traditional disinfectants like CHX. However, there are challenges in translating these findings into clinical practice, including the need for standardization of extract concentrations and ensuring the stability of active compounds, which can degrade over time. Future research should investigate the synergistic effects of different plant extracts or their combinations with established antimicrobials to enhance efficacy and broaden their spectrum of activity, while also addressing issues of formulation stability. Additionally, vitamin C contributes to reducing biofilm formation by supporting immune function and preventing bacterial adhesion. A deficiency in vitamin C can impair immune responses, promoting the development of biofilms (72). The roughness of materials also affects biofilm formation, as rough surfaces facilitate bacterial attachment, while smooth surfaces offer greater resistance (73). Therefore, combining adequate levels of vitamin C with smooth dental materials could improve the effectiveness of plant extract-based oral products.

In conclusion, all the tested extracts demonstrated a significant antimicrobial effect against anaerobic oral microorganisms. This finding indicates their potential use as mouth disinfectants in future clinical studies. Notably, the extracts from *L. stoechas*, *S. thymbra*, *S. parnassica*, and the hydroalcoholic extract of *V. myrtillus* were able to inhibit biofilm formation at concentrations of up to 1.25 mg/ml. This promising result suggests these extracts could be strong candidates for further clinical research. Additionally, future studies could explore the safety and efficacy of incorporating these extracts into long-term oral hygiene practices and their potential use in treating periodontal diseases.

## Data Availability

The original contributions presented in the study are included in the article/Supplementary Material, further inquiries can be directed to the corresponding author.

## Supplementary material

Supplementary Figure S1 HPTLC analysis of the hydroalcoholic extracts at 254 nm (panel **A**) and 366 nm (panel **B**).
