# Supplementary material for: Antibiofilm potential of plant extracts: inhibiting oral microorganisms and Streptococcus mutans
Source: Front Dent Med. 2025 Apr 4;6:1535753. doi: 10.3389/fdmed.2025.1535753 (PMC12006170; doi:10.3389/fdmed.2025.1535753)
Supplement: Supplementary Figure S1 — HPTLC analysis of the hydroalcoholic extracts at 254 nm (panel A) and 366 nm (panel B). [file Datasheet1.pdf]

Supplementary Figure 1

A

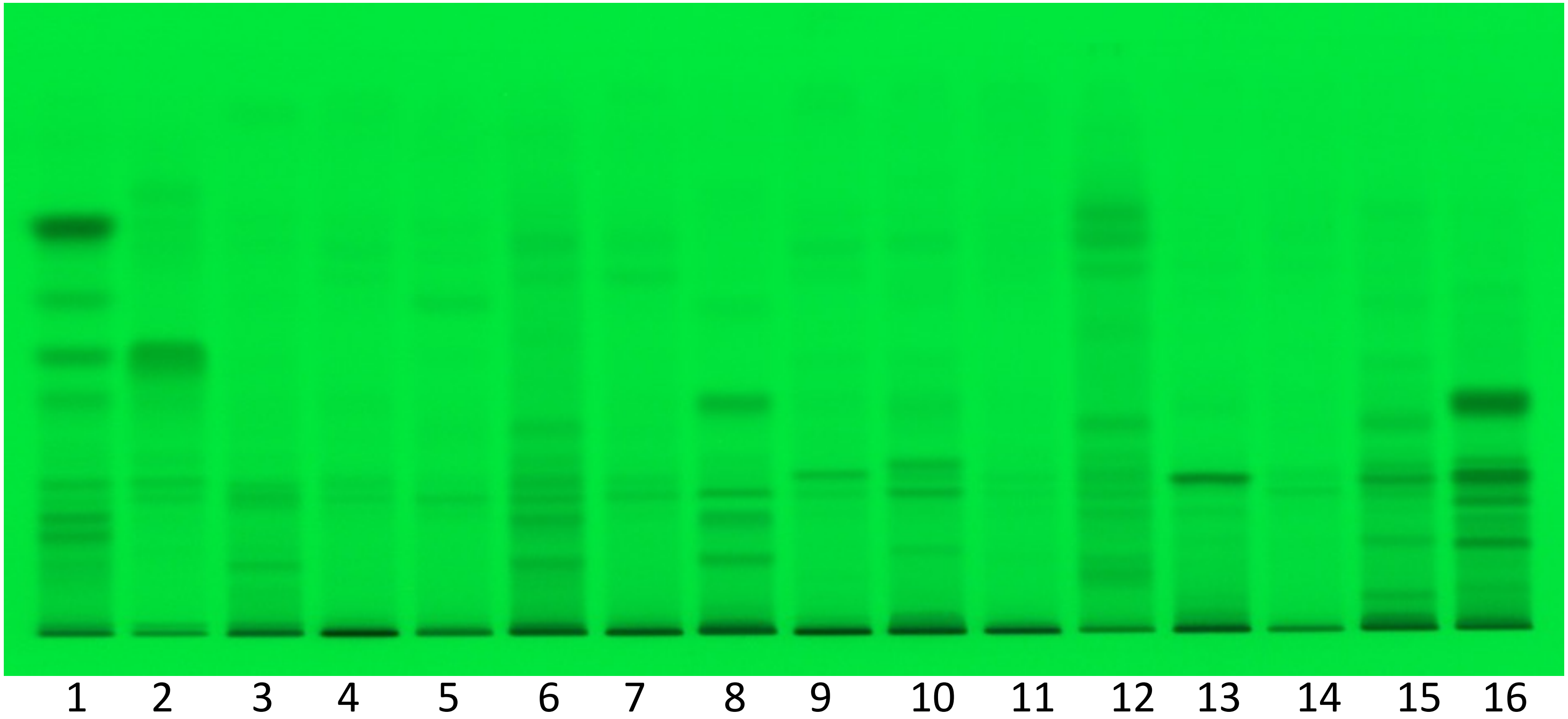

1: *M. longifolia*, **2:** *R. officinalis*, **3:** *L. stoechas*, **4:** *S. syriaca*, **5:** *M. aquatica*, **6:** *S. thymbra*, **7:** *S. parnassica*, **8:** *S. sclarea*, **9:** *S. euboea*, **10:** *S. spinosa*, **11:** *P. cretica*, **12:** *O. vulgare*, **13:** *A. taygetea*, **14:** *T. longicaulis*, **15:** *C. creticus*, **16:** *C. monspeliensis*

B

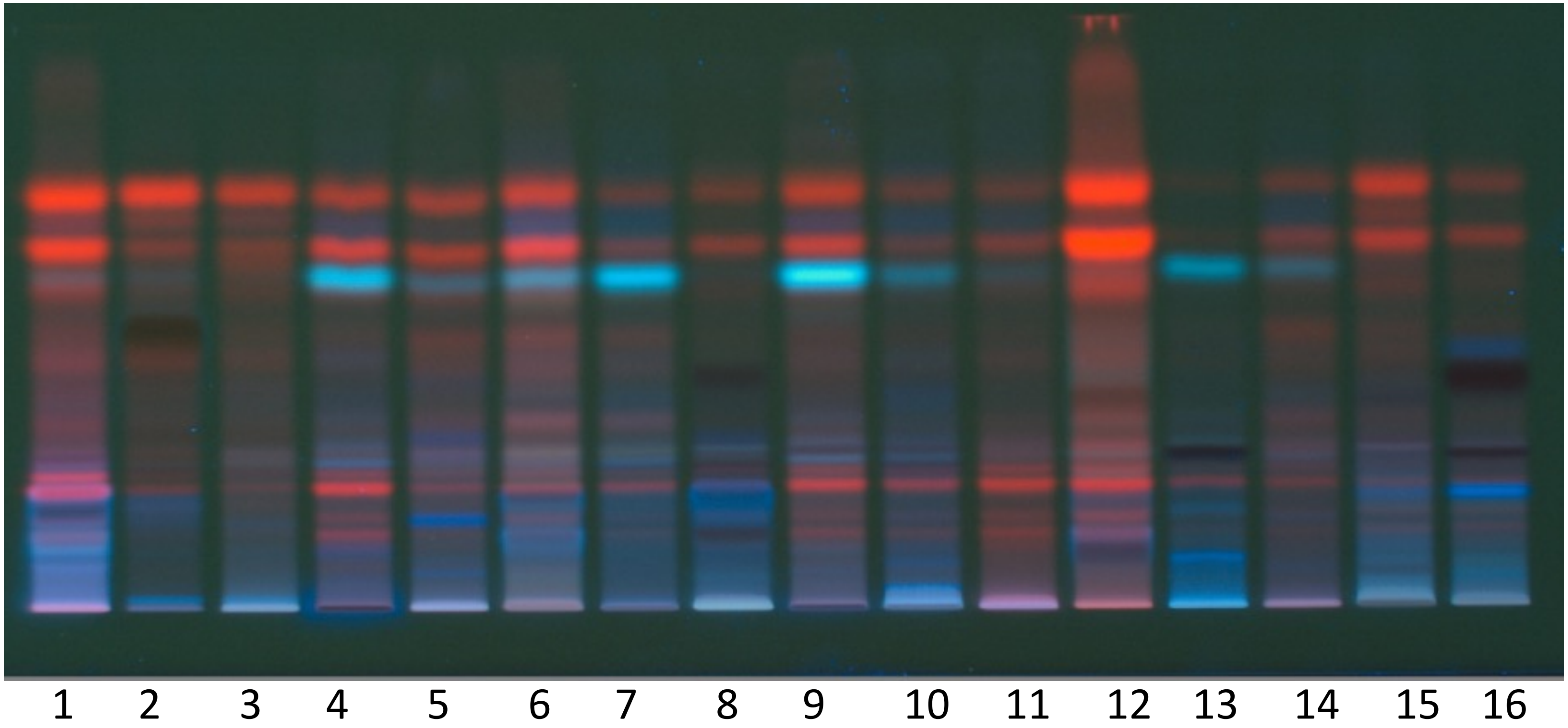

1: *M. longifolia*, **2:** *R. officinalis*, **3:** *L. stoechas*, **4:** *S. syriaca*, **5:** *M. aquatica*, **6:** *S. thymbra*, **7:** *S. parnassica*, **8:** *S. sclarea*, **9:** *S. euboea*, **10:** *S. spinosa*, **11:** *P. cretica*, **12:** *O. vulgare*, **13:** *A. taygetea*, **14:** *T. longicaulis*, **15:** *C. creticus*, **16:** *C. monspeliensis*
